# A *Mycobacterium ulcerans* vaccine pilot trial using an accurate low-dose challenge

**DOI:** 10.1128/spectrum.00555-24

**Published:** 2024-06-25

**Authors:** Stephen Muhi, Jessica L. Porter, Timothy P. Stinear

**Affiliations:** 1 Department of Microbiology and Immunology, Doherty Institute, University of Melbourne, Melbourne, Victoria, Australia; 2 Victorian Infectious Diseases Service, The Royal Melbourne Hospital, Parkville, Victoria, Australia; 3 WHO Collaborating Centre for Mycobacterium ulcerans, Victorian Infectious Disease Reference Laboratory (VIDRL), Doherty Institute, Melbourne, Victoria, Australia; National Center for Biological Sciences, Bangalore, India

**Keywords:** *Mycobacterium ulcerans*, infection model, *M. bovis* BCG, murine, challenge, vaccine, Buruli ulcer

## Abstract

A *Mycobacterium ulcerans* human challenge model has the potential to fundamentally advance our understanding of early human immune responses to infection, while rapidly evaluating vaccines and other therapeutic interventions. Here, using a murine tail infection model, we tested a very well-characterized working cell bank of the proposed challenge isolate *M. ulcerans* JKD8049 in naïve and *Mycobacterium bovis* bacille Calmette–Guérin (BCG)-vaccinated BALB/c mice. All 10 naïve mice were successfully infected with 20 colony-forming units (CFU) of *M. ulcerans* [95% confidence interval (CI) 17–22 CFU] with a mean time to visible lesion of 86 days (95% CI 79–92 days). In the 10 vaccinated mice, there was a significant delay in the mean time to lesion compared to the naïve controls of 24 days (*P* = 0.0003), but all mice eventually developed ulcerative lesions. This study informs a future human infection model by demonstrating the successful application of the challenge agent in this *in vivo* model and highlights both the promise and the problems with trying to induce protective immunity against *M. ulcerans*.

**IMPORTANCE:**

In preparation for its proposed use in a controlled human infection model (CHIM), this study reports the successful infection of BALB/c mice using a carefully characterized, low-dose inoculum of *Mycobacterium ulcerans* JKD8049 (our proposed CHIM strain). We also demonstrate that *Mycobacterium bovis* bacille Calmette–Guérin delays the onset of disease but cannot alter the course of illness once a lesion becomes apparent. We also validate the findings of previous low-dose challenges that used less accurate methods to determine the inoculum, but our presented methodology is practical, accurate, and anticipated to be reproducible.

## INTRODUCTION

*Mycobacterium ulcerans* is a slow-growing bacterium that causes the progressive, necrotizing infection of subcutaneous tissue known as Buruli ulcer (BU). BU is classified by the World Health Organization as a neglected tropical disease (1), emphasizing the importance of investing in the discovery of prevention and treatment strategies. The incidence of BU continues to increase in temperate regions of Australia, with cases recently reported in the state of New South Wales (2) and the metropolitan suburbs of major cities in the state of Victoria (3). Combination antibiotic treatment is typically lengthy, and minor side effects are common, although a cure without surgery is now the expected outcome for the vast majority of patients. In this context, we have previously proposed the concept of a controlled human infection model of Buruli ulcer (4), with the potential to accelerate the identification of a safe and efficacious BU vaccine. *Mycobacterium bovis* bacille Calmette–Guérin (BCG) has some evidence in humans of short-term protection against BU and remains the benchmark for efficacy testing of vaccines in murine vaccine models (5).

Research studies in the field of BU vaccination typically use *M. ulcerans* challenge doses that are very high, in the range of 10^4^–10^6^ colony-forming units (CFU) (5). However, our research has shown that a very low inoculum is sufficient to establish infection. The reported *M. ulcerans* ID_50_ (dose to infect 50%) is estimated to be just 2–3 CFU in BALB/c mice (6). This aligns with the likely mechanism of transmission in Australia, the mosquito (7), which is unlikely to transmit many bacteria given the low burden of *M. ulcerans* associated with individual mosquitoes (7). Previous mouse challenge experiments using such high inocula have, therefore, likely overwhelmed immunological responses with an unrealistically high infectious challenge, potentially underestimating the true efficacy of the vaccines tested. There are also possible differences between *M. ulcerans* isolates used in research settings, with African strains suggested to have properties such as thermotolerance (8) and express a more potent variant of the toxin mycolactone (9, 10), although the evidence supporting variation in strain pathogenic potential is not strong.

Previous studies of *M. ulcerans* “low-dose” challenge using needle puncture have relied on estimates of CFU count calculated using the measured surface area of a mouse tail, the surface area of a needle’s tip, the volume of culture media that adheres to the challenge site, and the concentration of bacteria in the media (measured 8–12 weeks later), while assuming that bacteria are evenly distributed over a mouse’s tail; this is despite the known propensity of the organism to form clumps, which further complicates accurate enumeration (6). These variables introduce numerous sources of uncertainty to the infectious dose calculation. A reproducible bacterial dose preparation method using a pre-defined and realistically low dose of *M. ulcerans* is a research priority for proposed human (4) and animal (11) infection trials. Using methods described elsewhere (12), we are now able to manufacture a working cell bank of *M. ulcerans* strain JKD8049 that is de-clumped, antibiotic-susceptible, non-chemically modified, mycolactone-producing, and stable after cryopreservation. The strain encodes the mycobacterial antigens that have been proposed as vaccine candidates (5) and is genetically stable during *in vitro* passage.

The overarching goal of the study presented here was to trial this characterized working cell bank of *M. ulcerans* JKD8049 in a mouse tail infection model that could inform subsequent human challenge trials. The specific aims of this pilot study presented here were to (i) demonstrate that *M. ulcerans* JKD8049 remains virulent, particularly after de-clumping, as aggregation is a reported virulence factor in other related mycobacteria (13); (ii) confirm the “attack rate” in BALB/c mice when challenged with ~20 CFU of *M. ulcerans* JKD8049; and (iii) demonstrate the clinical impact of any trained immunity provided by prior *M. bovis* BCG vaccination.

## MATERIALS AND METHODS

### Inoculum preparation

*M. ulcerans* JKD8049 was prepared and characterized as described previously (12). Quality control-tested cryovials were removed from −80°C storage and thawed on ice until no crystals were visible and then vortexed for 5 seconds. To confirm the final dose injected into mice, a 20-µL volume of a 10-fold dilution series of the suspension was spotted onto Middlebrook 7H10 agar (BD, Sparks, MD, USA) enriched with 10% oleic acid, albumin, dextrose, and catalase (OADC; HiMedia, Mumbai, India). The sample was then drawn into a 1-mL low dead space (LDS) syringe (B-Braun Omnifix-F, Melsungen, Germany); the sample volume was determined based on the CFU count from other vials produced in the same batch, aiming for a dose of 20 CFU/mouse, in a maximum final volume of 50 µL/mouse. A separate 1-mL syringe, containing phosphate-buffered saline (PBS) as a diluent, was connected to the syringe containing the sample using a luer lock connector (B-Braun Fluid Dispensing Connector, Melsungen, Germany). The sample was prepared in PBS by slowly plunging back and forth 15 times, after which the entire volume was pressed into a single syringe. The luer lock connector was removed and replaced with a 30G LDS hypodermic needle (TSK LDS-30013, Tochigi-Ken, Japan). The diluted sample was pressed to the tip of the needle prior to injection, and a new needle was used to inject each mouse.

### Vaccination and challenge procedure

The study used 6–8-week-old female BALB/c mice obtained from ARC (Canning Vale, Australia) and housed in individually ventilated cages, with food and water provided *ad libitum*. All mice were anesthetized with isoflurane during vaccination and challenge. *M. bovis* BCG (Danish strain 1331, AJ Vaccines, Copenhagen) was injected subcutaneously in the lateral aspect of the base of the tail (50 µL per dose). The *M. bovis* BCG dose was determined by spot plating the prepared vial on 7H10/OADC agar and incubating at 37°C for 6 weeks.

There were 14 mice in the vaccinated group comprising 10 animals for subsequent infectious challenge and 4 uninfected controls that each received a single subcutaneous dose of *M. bovis* BCG vaccine [53 CFU, 95% confidence interval (CI) 46–60 CFU]. A second group of 14 naïve mice received a media-only (sham) vaccination, comprising 10 animals for subsequent infectious challenge and 4 uninfected control animals for this group. Coinciding with the anticipated peak in host immune response to vaccination (14), infectious challenge was performed 8 weeks after vaccination or sham; 10 mice in each of the two groups were challenged with 20 CFU of *M. ulcerans* JKD8049 (95% CI 17–22 CFU). Negative control mice were injected with an identical volume of media only; Sauton’s media was used for sham BCG vaccination, and Sauton's media with Veggietones (SMVT) prepared as described previously (12) in 20% glycerol (vol/vol) was used for sham challenge.

**Fig 1:**
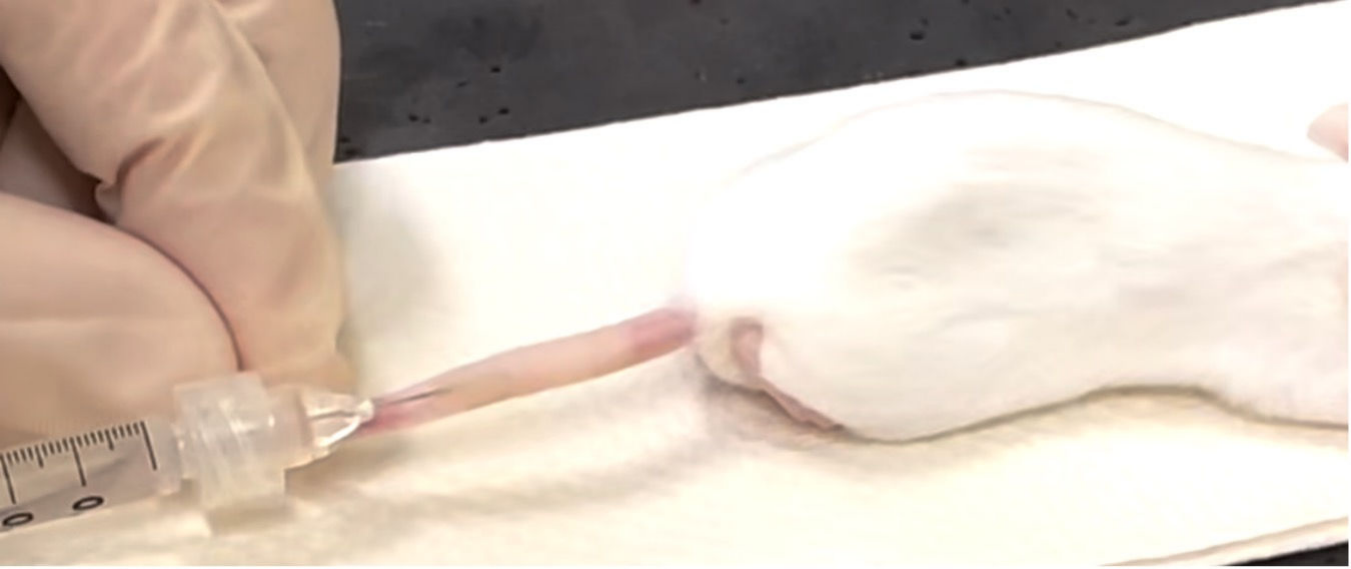
Subcutaneous tail injection of 50 µL of *M. ulcerans* into female BALB/c mouse. The needle was introduced, with the bevel facing up, near the dorsal aspect of the proximal third of the tail, between the lateral veins of the tail, and advanced approximately 3 mm prior to injection.

*M. ulcerans* and sham challenge were performed subcutaneously in the dorsal surface of the proximal tail, with the needle advanced approximately 3 mm proximally below the skin, using (and carefully avoiding) the lateral tail veins, which were convenient visible anatomical landmarks. The diluted challenge material was slowly injected in a 50-µL volume. After injection, a cotton swab was pressed firmly against the skin directly above the injection site, to minimize reflux as the needle was withdrawn. The challenge material was observed to cause superficial blanching of the skin from the injection site to ~1 cm from the entry point (Fig. 1), which resolved rapidly (≤24 hours). Mice were monitored twice weekly until a lesion was observed, followed by daily inspection thereafter. Mice were humanely killed by CO_2_ asphyxiation at the first sign of any skin ulceration.

### Tissue preparation, DNA extraction, and PCR

Mouse tissue was prepared for analysis as described previously (6). The ulcerated section of the tail was dissected using a scalpel, diced, and placed in a 2-mL screw-capped container with large glass beads (2 mm diameter, ~0.5 g). A 600-µL volume of PBS was added, and the tissue was homogenized by bead beating in a high-speed tissue disruptor (6,500 rpm) (Precellys 24, Bertin Technologies, France) with a total of four rounds of 2 × 30-second pulses; containers were placed on ice for 5 minutes between each round. Samples were stored at −80°C in PBS. DNA was extracted using a DNeasy PowerSoil kit (Qiagen). Control mice were included to monitor potential PCR contamination in addition to no-template negative PCR controls. IS*2404* quantitative PCR (qPCR) was performed using technical duplicates as described previously (15).

### Statistical analysis

Statistical analysis was performed using GraphPad PRISM (version 10.1.1). Continuous variables are reported as the mean/standard deviation or 95% confidence interval, as relevant. Comparisons between the groups were performed using Student’s *t*-test (for the difference between means) and illustrated using Kaplan–Meier curves using the log-rank test, with statistical significance if *P* < 0.05.

## RESULTS

### Mouse vaccination and infectious challenge

**Fig 2:**
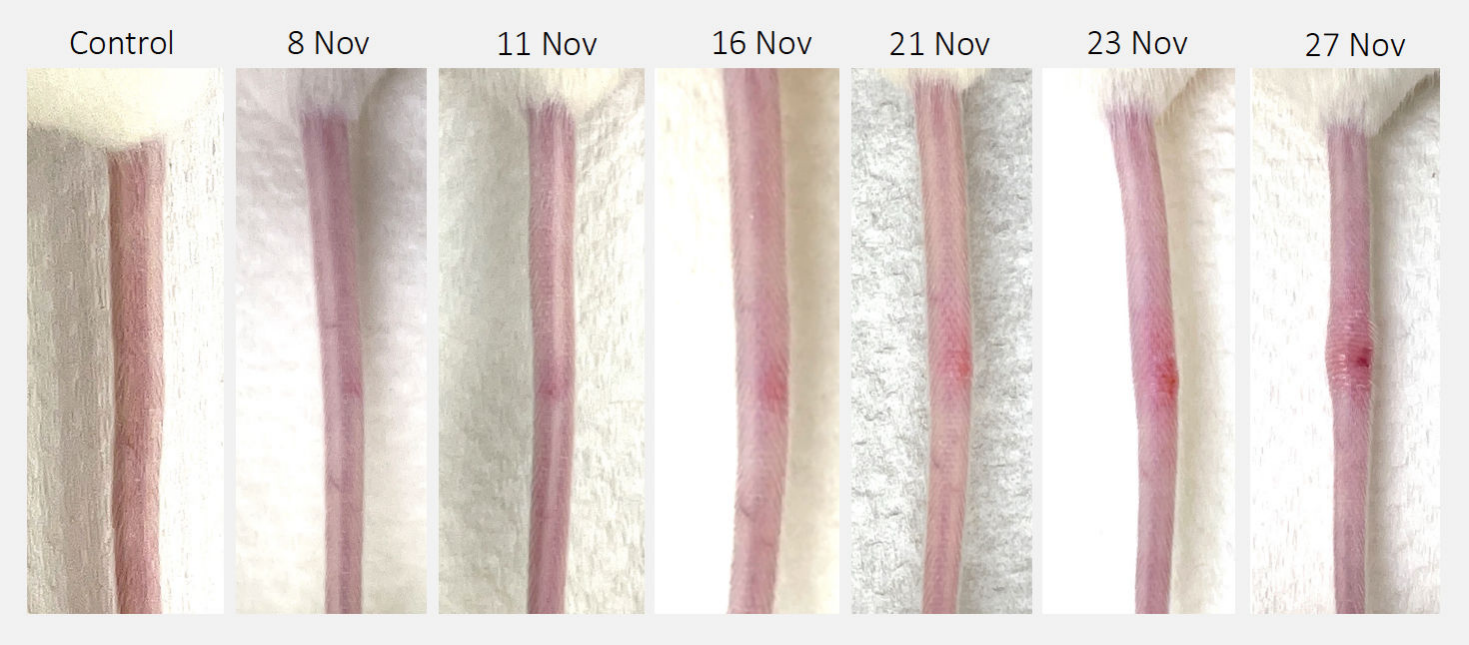
Typical progression of tail lesion in an unvaccinated female BALB/c mouse (#25.4) challenged subcutaneously with 20 CFU of *M. ulcerans* JKD8049. The initial lesion was first observed 3 months after challenge and progressed to ulceration after 30 days.

**Fig 3:**
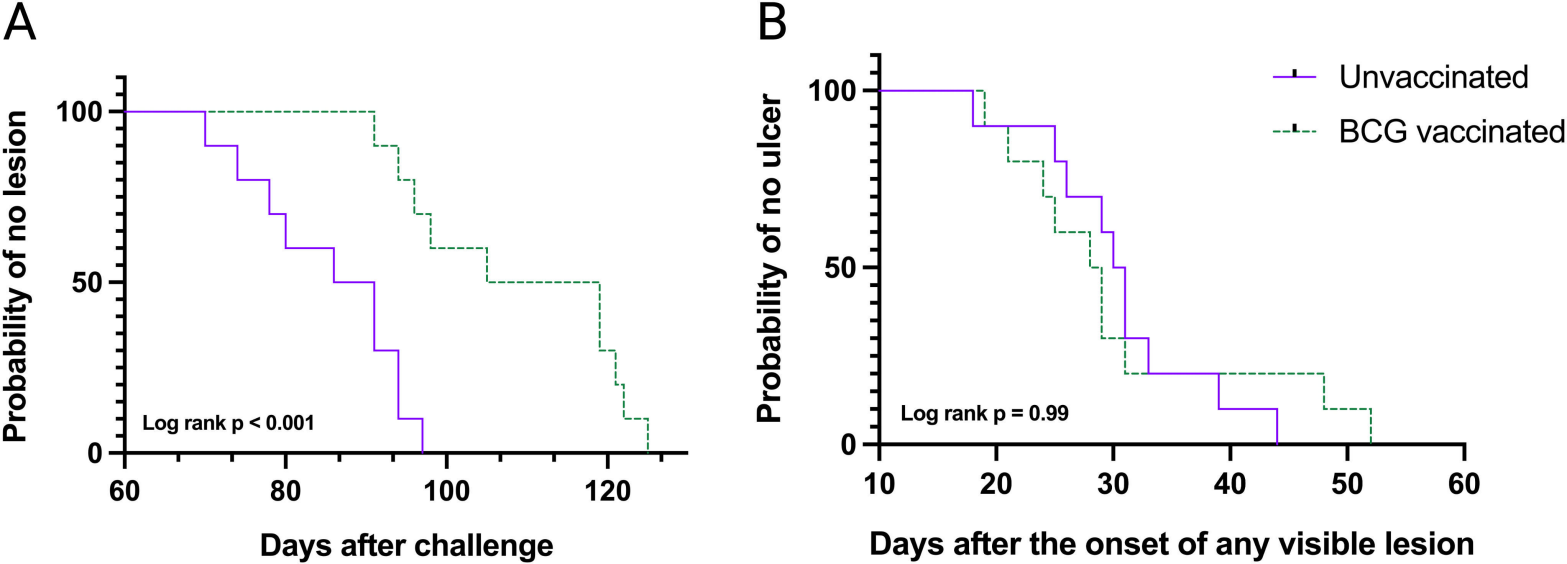
Kaplan–Meier curves for female BALB/c mice challenged with 20 CFU of *M. ulcerans* JKD8049 (see Table S1). (**A**) Time-to-lesion onset after challenge in *M. bovis* BCG-vaccinated and naïve mice. (**B**) Time-from-lesion onset to ulceration in *M. bovis* BCG-vaccinated and naïve female BALB/c mice.

All 20 mice challenged with 20 CFU of *M. ulcerans* JKD8049 developed characteristic lesions at the inoculation site, which eventually developed into ulcerative lesions (Fig. 2). In unvaccinated mice, we observed a mean time-to-lesion onset of 86 days (95% CI 79–92 days), and in vaccinated mice, the average time-to-lesion onset was 109 days (95% CI 99–119 days) (*P* = 0.0003) (Fig. 3A). Despite these lesions, mice were otherwise healthy and did not appear to groom or attend to the lesions. There was no significant difference in weight change in BCG-vaccinated infected mice compared to controls (Student’s *t*-test, *P* = 0.95) or infected naïve mice compared to controls (Student’s *t*-test, *P* = 0.83) (Table S1). No distant lesions were observed beyond the initial injection site in any mouse. None of the four sham-challenged mice vaccinated with *M. bovis* BCG, nor the four mice given media only, developed lesions by the end of the 6-month experimental period.

Once lesions became visible, there was no significant difference in the subsequent time to ulceration, with mean 31 days in both groups (95% CI 23–38 days in vaccinated mice and 25–36 days in unvaccinated mice) (Fig. 3B). This suggests that any trained immunity induced by vaccination in BALB/c mice delays progression of infection, but only until the stage where tail lesions become clinically obvious.

### IS*2404* qPCR of tail lesions confirm infection

To establish that tail lesions were due to *M. ulcerans* infection, IS*2404* qPCR was performed on 12 tail homogenates, four of which were vaccinated and challenged, four naïve and challenged mice, and two in each control group. This analysis confirmed that mice challenged with *M. ulcerans* JKD8049 were all IS*2404* qPCR-positive (mean cycle threshold 18, 95% CI 16–21), and unchallenged mice were qPCR-negative (Table S1).

## DISCUSSION

This pilot study confirms that the working cell bank of *M. ulcerans* JKD8049 produced for this trial contained virulent bacteria and was able to infect all mice with 20 CFU of *M. ulcerans*, demonstrating the accuracy of the bacterial dose and sensitivity of the infection model. Notably, we reproduced the incubation period of 12 weeks (range 10–14 weeks) reported in prior low-dose studies where the inoculation dose was estimated rather than directly measured (6). Compared to earlier studies, we also observed a high attack rate, with the precise low dose prepared here infecting 20/20 mice (100%) compared with 21/24 (88%) (6) and 9/10 (90%) (16).

This report also concurs with prior research that demonstrated *M. bovis* BCG vaccine offers protection against a low dose of *M. ulcerans* (16), although long-term immunity was not demonstrated. The dose of *M. bovis* BCG we used here was lower than anticipated, as reconstituted vials of this commercial product should contain 2–8 × 10^5^ CFU/mL. It is possible that the *M. bovis* BCG CFU count we obtained might be an underestimate, as no mycobacterial de-clumping was performed prior to spot plating, and bacterial viability may have declined between reconstitution and vaccination. Nevertheless, previous studies support the use of low doses of *M. bovis* BCG vaccine to protect against experimental tuberculosis aerosol challenge in BALB/c mice, although predominantly cell-mediated (T_h_1-biased) responses occur at such low vaccine doses (17). Additionally, *M. bovis* BCG doses used in mouse vaccination trials are typically like the doses used to vaccinate humans (10^4^–10^7^ CFU) (5), despite female BALB/c mice weighing ~20 g. If the estimated *M. bovis* BCG dose used in the present study was accurate, then it is approximately the weight-adjusted equivalent dose for a human 70 kg in weight (~2.5×10^3^ CFU/kg). Future experiments using a higher dose of *M. bovis* BCG are needed to better assess our low-dose *M. ulcerans* infection model. This study also emphasizes that long observation periods after challenge (>6 months) may be necessary to capture the development of a lesion during a potentially extended incubation period.

Although immunological responses to low-dose infection have been reported previously (16), future work will aim to examine responses to a range of low-challenge doses and in more diverse mouse strains (18). While mice are not humans, a comprehensive interrogation of murine immunological responses in genetically diverse animals to low-dose *M. ulcerans* infections might better predict the range of clinical and immunological responses in humans (14). We did not observe a self-healing phenotype in this study, although our humane end-point for euthanasia was the first sign of skin ulceration; it is possible that some mice may have healed spontaneously if allowed to progress further, although this is not typically observed in BALB/c mouse models of BU (5).

The incubation period identified in this low-dose model is remarkably like natural infections in humans (4–5 months) (19), despite the considerably thinner subcutaneous layer and other tissue differences between mice and humans. The efficacy of this *M. ulcerans* infectious challenge dose is further progress toward developing a controlled human infection model. Considering the 100% attack rate we observed with 20 CFU in 20 mice, a formal low-dose LD_50_ experiment could be performed using this murine infection model.

## SUPPLEMENTAL MATERIAL

Table S1 spectrum.00555-24-s0001.docx Dates of lesion appearance and ulceration, and weights immediately prior.

ASM does not own the copyrights to Supplemental Material that may be linked to, or accessed through, an article. The authors have granted ASM a non-exclusive, world-wide license to publish the Supplemental Material files. Please contact the corresponding author directly for reuse.
