# Supplementary material for: A Mycobacterium ulcerans vaccine pilot trial using an accurate low-dose challenge
Source: Microbiol Spectr. 2024 Jun 25;12(8):e00555-24. doi: 10.1128/spectrum.00555-24 (PMC11302252; doi:10.1128/spectrum.00555-24)
Supplement: Table S1 — Dates of lesion appearance and ulceration, and weights immediately prior. [file spectrum.00555-24-s0001.docx]

**Table S1:** Dates of lesion appearance and ulceration, and weights immediately prior to lesion onset and at the time of visible ulcer, following challenge in naïve and BCG-vaccinated BALB/c mice challenged with 20 CFU of *M. ulcerans* JKD8049.

*Negative control mice remained free from any visible lesion; representative dates were selected to serve as a comparison to experimental mice. Ct: cycle threshold.
